# In Others' Shoes: Do Individual Differences in Empathy and Theory of Mind Shape Social Preferences?

**DOI:** 10.1371/journal.pone.0092844

**Published:** 2014-04-17

**Authors:** Florian Artinger, Filippos Exadaktylos, Hannes Koppel, Lauri Sääksvuori

**Affiliations:** 1 Warwick Business School, University of Warwick, Coventry, United Kingdom; 2 Max Planck Institute for Human Development, Berlin, Germany; 3 BELiS, İstanbul Bilgi University, İstanbul, Turkey; 4 University of Heidelberg, Heidelberg, Germany; 5 University of Hamburg, Hamburg, Germany; George Mason University/Krasnow Institute for Advanced Study, United States of America

## Abstract

Abundant evidence across the behavioral and social sciences suggests that there are substantial individual differences in pro-social behavior. However, little is known about the psychological mechanisms that underlie social preferences. This paper investigates whether empathy and Theory of Mind shape individual differences in pro-social behavior as conventionally observed in neutrally framed social science experiments. Our results show that individual differences in the capacity for empathy do not shape social preferences. The results qualify the role of Theory of Mind in strategic interaction. We do not only show that fair individuals exhibit more accurate beliefs about the behavior of others but that Theory of Mind can be effectively used to pursue both self-interest and pro-social goals depending on the principle objectives of a person.

## Introduction

Abundant evidence across the behavioral and social sciences suggests that there are substantial individual differences in pro-social behavior. While some people predominantly care about their own material outcome, other people with more pro-social motivations are often seeking to advance social goals and equality at their own cost. These insights, often supported by laboratory experiments, have had a decisive impact on the emergence of social preference theories [1]–[3]. Yet, to date it is unclear which psychological mechanisms underlie the observed behavior.

Empathy and Theory of Mind (ToM) are both regarded as central in social interaction relating to social emotions and social reasoning. Social emotions function as emotional responses to, for instance, unfair or fair decisions. Social reasoning is used to assess how others are likely to act in a given situation. Empathy is thereby the capacity to *share social emotions* of others. ToM is the capacity to *understand* the social reasoning and social emotions of others [4].

Particularly prominent in the study of pro-social behavior and individual differences in social preferences are the Ultimatum Game (UG) [5] and the Dictator Game (DG) [6]. These paradigmatic games are regularly implemented to study decision making in neutrally and socially framed contexts. Studies have examined decision making using the UG and DG in various social contexts, for instance, by the means of displaying the face of the experimental participant before the interaction [7], [8]. Likewise, a vast array of studies has examined behavior in the UG and DG using a neutral decision context. The conventional practice of using neutral and abstract frames in economic experiments is a means to improve experimental control and elicit the underlying preferences in a given population. Using a neutral frame, the two games have been, for instance, employed in the parameterization of social preferences in the inequity aversion model by Fehr and Schmidt (1999), currently the most widely cited model on social preferences [2].

In the UG, a proposer chooses how much to offer from an initial endowment. A responder can either accept or reject. If the responder rejects, both will get nothing. In the DG, the responder must accept any offer made by a proposer. With its absence of strategic considerations, the DG is often regarded as a pure measure of pro-social behavior and hence lends itself to evaluating the role of empathy and social preferences [6]. Regarding the UG, across a large range of conditions, responders have been shown to reject low offers despite the consequence of receiving a payoff of zero [9]. These rejections have often been interpreted as evidence for social preferences (for alternative interpretations of UG rejections see [10], [11]). Given that a substantial share of responders in the UG will reject low offers, many inherently selfish proposers make apparently fair offers in order to maximize their expected pecuniary payoff. At the same time, those individuals better at forecasting what is the smallest offer still accepted by the receiver can use this knowledge to their material benefit. Because of its combination of strategic interaction and fairness concerns, the UG is particularly well suited to evaluate the role of ToM in strategic interaction.

This paper tests whether individual differences in empathy and ToM affect behavior in neutrally framed experiments which are frequently employed to measure social preferences. Scholars of cognitive science have stressed the importance of embodiment in *emotion recognition* and posit that ability to understand social emotions is grounded in primitive ability to share emotions through bodily interaction with other people and the environment [12]. Our study differs from the literature on embodied cognition in that we investigate the underlying psychological mechanism of social preferences in a neutrally framed context. This renders it possible to directly evaluate the role of empathy and ToM in social preference theories that are used for instance to predict economic behavior, analyze welfare implications and derive policy recommendations.

Our results show that individual differences in the capacity for empathy, as measured through various psychometric tests, do not shape social preferences. At the same time, our results qualify the role of ToM in strategic interaction. We do not only show that fair individuals exhibit more accurate beliefs about the behavior of others but that ToM can be used to pursue both self-interest and pro-social goals depending on the principle objectives of the person.

### Empathy

Looking inside the black box of social preferences, one component that has been hypothesized to play a central role is empathy, the capacity to share the feelings of others. Adam Smith (1789, p. 10) already highlighted in *The Theory of Moral Sentiments* the potential role of empathy in pro-social behavior: “Empathy is the source of our fellow-feeling for the misery of others, that is by changing places in fancy with the sufferer, (it is) that we come either to conceive or to be affected by what he feels” [13].

More recently, a very similar stance has been taken in the perception-action model of empathy [14]. The model suggests that it is sufficient to observe or imagine someone else in an emotional state to trigger an empathic response. In a review article on the role of empathy and ToM in economics, the authors suggest that if empathy implies the shared experience of emotions, this can undermine the idea that choices are based solely on self-interest [15]. This is captured, for instance, by the inequity aversion model by Fehr and Schmidt (1999), in which an agent can experience not only positive but also negative utility if the agent's own and others' monetary outcomes differ [2]. In line with this reasoning, empathy has been listed among a number of pro-social emotions, such as guilt and shame, which underpin pro-social behavior in human decision making [16].

Individual differences in the capacity for empathy reflect differences in pro-social behavior in domains such as volunteering and donating [17]. In a group of young adults, measures of pro-social dispositions have been found to be stable across a period of five years and relate to ratings of empathy [18]. According to the empathy-altruism hypothesis, empathy is even regarded as the exclusive source of genuine altruism [19]. Empathic feelings are classically associated with helping someone in need [20], [21]. In the context of the Prisoner's Dilemma Game, inducing empathy via portraying the other person as needy resulted in participants choosing to cooperate more often than in a neutral control condition even when participants knew beforehand that the other person would defect on them [22].

Neuroscientific experiments have indicated that people differ in their empathic capacity and that this might relate to individual differences in social preferences [23]. Inviting couples into the laboratory, participants received a painful stimulus and the resulting brain signal was compared to that obtained when they were merely informed that their loved one had received such a stimulus [24]. The same affective brain circuits were active both when receiving pain and when being informed about the beloved one experiencing the same painful stimulus. Importantly, the higher the activation in the pain circuits, the higher participants scored on a psychometric measure of empathy, the Interpersonal Reactivity Index, which is also employed in the current investigation [25]. In a further study participants were initially exposed to a situation where they were treated either fairly or unfairly by a matched partner [26]. In the second part of the experiment, the matched partner received a painful stimulus. For men, empathy-related brain activity was found only in those who faced a fair partner and not in those who faced an unfair partner. For women, such a difference was not observed. The authors link these results on empathy to social preference theories, where people value others' gains positively if they are fair, but the gains of unfair partners are negatively valued.

A number of researchers have hypothesized about the importance of individual differences in empathy for fairness as observed in neutrally framed laboratory games [15], [27], [28]. If allocators were explicitly asked to put themselves in the shoes of the recipient in the DG, offers increased as compared to individuals in a control group that were not given any specific instructions [28]. This illustrates that empathic behavior can be induced by changing the social context. In this study, we specifically focus on individual differences in empathy in a given context and how these relate to social preferences. Singer (2008, p. 264) reemphasizes a point made earlier by Singer and Fehr (2005) that “one prediction that can easily be made is that people with a greater ability to empathize should display more other-regarding behavior” [4], [27]. However, a clear-cut demonstration that individual differences in empathy underlie social preferences as classically measured in neutrally framed laboratory games is still missing.

*Hypothesis 1: The greater the individual's capacity of empathy the higher the offer in the DG.*

### Theory of mind

Empathy relates to the capacity to *share* social emotions. In contrast, ToM allows an agent to *understand* the social reasoning and social emotions of others. Utilizing ToM, the decision maker constructs the mental states of others making inferences about beliefs, intentions, and emotions [29]. The concept of ToM was initially proposed by primatologists who suggested that it emerged as a result of the social challenges of living in larger coalitions [30], [31].

In economics, Smith (1776) noted in *The Wealth of Nations* that understanding the goals and beliefs of one's trading partner facilitates business [32]. The centrality of ToM to strategic interaction implies that it closely relates to issues addressed in game theory. One basic assumption is that of “common knowledge,” which implies that interacting agents reflect on the action of the other and know that the other does the same [33]. More recently, the assumption that all people make the same inferences as others do has been challenged, and individual differences between agents in their thinking steps and hence in their capacity for ToM have been considered central to explaining a number of empirical phenomena [34], [35]. This echoes the hypothesis that Singer and Fehr (2005) postulated, by which individuals who have a higher capacity for ToM can better predict others' motives and actions [27].

*Hypothesis 2: The higher the individual's capacity for ToM the more accurate the stated beliefs about the behavior of others.*

ToM is thought to serve two functions: (i) It facilitates the pursuit of one's personal gains, a function that in the psychological literature has also been labeled Machiavellian intelligence [30]; and (ii) it facilitates pro-social behavior, as shown, for instance, in non-human primates where the capacity for ToM can lead to acts of spontaneous helping [36]. Surprisingly, to date, among humans there exists evidence only for the second function but not for the first. For instance, people high on the Machiavellian scale score low on the capacity for ToM, that is they do not seem to effectively employ ToM to further their own goals [37], [38].

Using economic games, researchers have studied individual differences in ToM among children but not adults: In the UG, 6- to 10-year-old autistic children, who are impaired in ToM, were more likely to accept low offers and to refuse fair proposals compared with a normally developed cohort [39]. Likewise, preschoolers who had acquired the capacity for ToM made higher mean offers in the UG than those who had not yet developed this capacity. This has led to the proposition that individual differences in ToM are a psychological component that underlie social preference theories in which those with a higher ToM are fairer [40].

Yet, these findings do not square with the proposition made in economics and game theory that those who seek to maximize their own monetary profit can benefit from an accurate estimation concerning others' likely action. The seminal study of Kelley and Stahelski (1970) on the role of beliefs in social dilemmas allows a reconciliation of these contradictory propositions [41]. The authors showed that cooperative individuals are more accurate than selfish individuals in predicting the actions of others in a social dilemma. They argued that this difference is functional in nature: To pursue their strategy successfully and reduce the likelihood that someone else takes advantage of their pro-social tendency, cooperative people need more accurate beliefs than defectors. For defectors it is unnecessary to make accurate inferences in a one-shot social dilemma, as doing so does not affect their strategic choice which is always to defect regardless what the other does. For those that consider cooperating, it is vital to accurately estimate what others do in order to be able to deduce the potential risk that is entailed in choosing to cooperate. A question that follows from this is whether people that tend to favor a strategy with a pro-social outcome are generally more accurate in estimating what others do regardless the specific context of the game. We used the DG in an attempt to distinguish the principal social preferences of agents, that is, whether the agents seek to maximize their own payoff or also care about the payoff of others.

*Hypothesis 3: When comparing fair and selfish participants as measured by their offers in the DG, fair participants will have more accurate beliefs about the decisions of other participants than selfish ones.*

However, the situation changes when the focus is on those proposers in the UG who seek to maximize their own payoff. The more accurately a proposer judges the likelihood of a certain offer being rejected, the higher the expected payoff. Hence, in this context it becomes functional for the selfish proposers to have accurate beliefs.

*Hypothesis 4: Selfish participants, as measured by offers in the DG, are more likely to employ accurate beliefs for their own material self-interest in the UG than fair participants.*

## Methods

The experiment was conducted at a German University where ethical review is standardized for conventional socioeconomic experiments such as this one. This implies that the treatment of participants was in agreement with the ethical guidelines of the German Psychological Society (DGP - see the guidelines: http://www.bdp-verband.org/bdp/verband/ethic.shtml; particularly section C.II.4) and the German Research Foundation (Deutsche Forschungsgemeinschaft). Specifically, all participants gave their written informed consent to participate voluntarily, assuring them that analyses and publication of experimental data would be without an association to their real identities. Moreover, random assignment to visually separated cubicles and private payment at the end of the experiment preserved the anonymity of participants. The experiment involved no deception of participants. As in other socioeconomic experiments, there were no additional ethical concerns.

The experiment was computerized and conducted in the laboratory of a large German University using z-Tree [42]. Participants were recruited via the online recruitment system ORSEE [43]. A total of 120 students took part in one of four experimental sessions. The vast majority of 87 female and 33 male participants were undergraduate students representing a wide range of different academic disciplines. We did not aim to recruit any specific gender composition. The academic disciplines of the participants were as follows: 31 social sciences (excluding economics), 22 natural sciences, 15 humanities, 12 law, 12 economics and business administration, 6 medicine and 18 other disciplines. The reported distribution of academic disciplines includes 116 individuals. We have excluded from the data set four non-native German speakers who indicated limited ability to understand some parts of the experimental instructions originally written in German. Each experimental session lasted about two hours. Earnings per participant ranged from 7€ to 26€ with a mean of 14€.

### Behavioral measures

The behavioral measures of our experiment consist of three separate sections. The first section includes three decisions to measure individuals' concern for fairness. The second section elicits participants' belief about the behavior of other participants in the first section. The third section measures participants' risk attitude using incentivized risk elicitation task. Decisions in all sections are incentivized by randomly selecting one decision from each section for the final payment (please see the Instructions S1 for the Experimental Instructions).

To measure an individual's concern for fairness, we had participants make decisions in three different roles: As a proposer in the UG and in the DG and as a responder in the UG. When participants must indicate their actions in all possible roles this has been labeled the strategy vector method [44]. When the strategy vector method has been compared to situations where participants play only one role in bargaining experiments, no major differences has been found [45]. These previous results lend support to the argument that individuals' concerns for others as measured in laboratory experiments are robust to elicitation through the strategy vector method. In the role of the responder, participants indicated the minimum acceptance level below which they would reject an offer. At the end of the experiment, one of the three decisions was picked randomly to calculate the monetary payoff. Participants were not informed whether they were matched with a different participant in each decision. This feature may in principle affect the behavior of various individuals.

In both games, the proposer was endowed with 90 experimental currency units (ECUs) and could split these in intervals of 10. An equal split is known to be the modal offer both in the UG and the DG. Hence, our research design precluded the possibility of making the modal offer. It has been shown that after removing the equal split, fair offers become less frequent [46] and that responders are less averse to unequal outcomes [47]. This provides a particularly interesting test bed for individual differences in ToM.

Participants were additionally asked to indicate their beliefs about the likely action of a randomly chosen partner in each of the three decision tasks (DG offer, UG offer, and UG minimum acceptance level). They thereby stated the probability they considered most likely for a given action by answering the question such as the following example for DG offers: “Please indicate the likelihood that a randomly determined person taking part in this experiment has chosen one of the 10 possible divisions”. Beliefs about the actions of others were rewarded based on the Quadratic Scoring Rule (QSR) [48]. It is used to measure and reward the accuracy of predictions. In our case, each participant states a probability vector *r* = (*r*_1_,…,*r_n_*) where *r*_i_ refers to the probability that an event *i* occurs. The QSR is then used to give a reward of Q(r,j) where *j* is the event that actually occurs. The functional form of the QSR
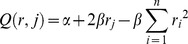
is strictly concave with respect to *r_i_* in which case the highest possible earning is received by placing 100% on the occurring event and zero probability on any other event. Participants get penalized for placing a positive probability on events that actually do not occur. The penalty is disproportionately increasing in the probabilities placed on those events. We set α = 10 and β = 10 which guarantees a positive payoff unless the participant assigns 100 per cent probability on a single false event in which case the participant receives no payment. An empirical assessment of various proper scoring rules (logarithmic, quadratic and spherical) show that participants are likely to employ various suboptimal reporting strategies when the score contains both positive and negative scores [49]. In our experiment, we have limited the QSR score to positive scores except in case where 100 percent probability is assigned to a single false event to facilitate truthful revelation. Schotter and Trevino [50] present a recent survey of the literature on belief elicitation in laboratory experiments and discusses among other techniques the QSR. Our implementation of the rule closely follows the standards for incentive compatible belief elicitation in economic experiments.

The QSR rule induces truthful revelation of subjective beliefs about the expected behavior of other participants in strategic games and is widely used to elicit beliefs in behavioral experiments [51]. To ensure full comprehension of the payoff mechanism, participants were carefully instructed on the procedure. Each participant had to go through three learning episodes with control questions that became increasingly difficult. A post-hoc analysis of the data shows that 93 per cent of participants provided the right answer to all three questions. At the same time, 96 per cent of participants provided the right answer to the most complex question (Question 3). However, a considerable effort and learning through trial and error was needed to gain full comprehension. Had we allowed only one attempt per question, only 52 per cent of the participants would have been able to find the right answer to all three questions.

For the purpose of paying, the QSR score for each participant in a given task was calculated on the basis of how well stated beliefs predicted the decision of another, randomly selected, participant. In order to make a robust assessment of the accuracy of beliefs and their relation to social preferences in the results section of the paper, the reported belief distribution of each participant was compared to the distribution of choices of all other participants. The belief assessment of every participant was matched with each of the observed outcomes in the study population, resulting in 115 scores, as there were 116 participants in total. We then computed for every participant a mean score for the three separate tasks and an overall score that is aggregated over the three tasks. These scores could range from zero to two. A score of zero indicates that the participant assigns 100 percent probability to a single false outcome. A score of two indicates that the participant assigns 100 percent probability to a single correct outcome.

When beliefs and actions from the same participants are elicited in an incentive-compatible manner, participants might hedge across tasks that are independently incentivized. In other words, participants might take in some task higher risks to increase their chance of higher earnings and compensate for this in other tasks where they take lower risks ensuring that they get a minimum payoff. Previous studies however show that unless hedging opportunities are very prominent, results are unlikely to be confounded due to hedging [52]. Because participants did not know about the belief elicitation until they were actually asked to state their beliefs, the prominence of hedging opportunities between the games and belief elicitation tasks was not an issue here. Eliciting a separate belief distribution in each game may encourage participants to balance their reported beliefs. For example, participants could report systematically wider belief distributions in a certain game to secure a high minimum payment if this task gets chosen for the payment. As we only paid one randomly chosen belief elicitation task, such hedging should be minimized.

A possible remaining confound is that participants adjust their reported beliefs to their risk attitude. Likewise, the practice of paying participants based on the action chosen by one randomly determined person may affect the reported belief distributions. This may invite risk-seeking participants to report narrower distributions centered on the option that they believe to be the most likely event. Risk-averse participants may be inclined to report flatter distributions to secure a high minimum payment. Not only beliefs but also behavior in the games might be shaped by individual differences in risk aversion. Greater risk aversion can, for example, lead to higher offers of proposers in the UG as these are less likely to be rejected.

In order to control for individual differences in risk aversion regarding the stated beliefs and decisions in games we used the Holt-Laury lottery task [53]. This is an incentive-compatible task where participants choose 10 times between two different lotteries that gradually vary the combination of probabilities and monetary outcomes in order to measure each individual's degree of risk aversion.

### Psychometric measures

Individual differences in the capacity for ToM and empathy have traditionally been assessed using psychometric tests. We used two tests that both measure empathy and ToM. The two empathy measures are convergent, whereas the two ToM measures complement each other: (i) “Cold” ToM is about inferences regarding the epistemic state of others and refers to the knowledge, beliefs, and intentions that someone else holds; (ii) “hot” ToM is about inferences about others' emotions [54].

To measure cold ToM and empathy, we employed the Interpersonal Reactivity Index (IRI),which is the most widely used psychometric test to evaluate both empathy and ToM. The test has been extensively investigated and validated [25]. The German translation of the IRI, the Saarbrücker Persönlichkeits-Fragebogen [55] was used in the experiment. The IRI is a self-report questionnaire using abstract descriptions of social interaction which participants respond to. The modified version we used had four dimensions, each containing four statements. Two dimensions were used for the final analysis: Cold ToM was measured using the perspective-taking scale where participants responded to statements such as, “I try to look at everybody's side of a disagreement before I make a decision.” The capacity for empathy was measured with the scale for “empathic concern”. Participants had to respond to statements such as, “I often have tender, concerned feelings for people less fortunate than I.” Individual differences in the empathic concern scale correlate positively with brain activity associated with empathy [24]. Participants indicated the extent to which a statement described them on a 5-point scale, from *does not describe me well* to *describes me very well*. Cronbach's α was .*73* for the ToM measure and .*80* for the empathy measure, which correspond to the values found by Davis (1980) [25].

A potential problem with psychometric tests, in particular regarding participants that are primarily motivated to increase their own earnings, is that responses are not incentivized. Specifically, Lyons, Caldwell, and Shultz (2010) point out that this might be problematic when it comes to assessing the relationship between ToM and Machiavellianism [38]. In their experiment, participants high on the Machiavellian scale showed a lack of effort in the ToM task, indicating that they might not have been sufficiently motivated by non-incentivized tests to reveal their ToM level. Note that measuring how accurate a participant's believes are what others will do in the games should be equivalent to how accurate a person is on inferring the epistemic state of someone else. The former is assessed using the QSR and is incentive compatible, the latter is assessed using the psychometric scales for ToM.

To measure hot ToM and empathy, we used the Multifaceted Empathy Test (MET), which employs 40 realistic photographs of faces expressing positive or negative emotions as stimuli [56]. The MET was originally developed to measure individual differences of empathy and ToM for people with autism as these have difficulties with abstract descriptions employed for instance by the IRI. The test is also suitable to measure individual differences in a normally developed cohort. It uses pictures as stimuli. Participants answered for each picture three types of questions reflecting three subscales. The subscale emotion recognition measured hot ToM by assessing to what extent participants could correctly infer the emotional state of others as depicted in the photographs. Participants answered the question, “What does this person feel?” by selecting one of four possible options, where only one was correct. A similar test for hot ToM, relying on emotion recognition in faces, was used by Paal and Bereczkei (2007) and Lyons, Caldwell, and Shultz (2010), who found a positive relationship between cooperativeness and the capacity for ToM [37], [38]. To measure empathy, the Multifaceted Empathy Test provides two subscales: The subscale *direct empathy* asked participants to answer the question, “How strongly do you feel with this person?” The subscale *indirect empathy* asked the question, “How aroused are you by the picture?” Direct and indirect empathy were measured with a 9-point scale ranging from *not at all* to *a lot*. Cronbach's α was .*95* for direct empathy, .*96* for indirect empathy, and .*70* for hot ToM, which correspond to the values found by Dziobek et al. (2007) [56].

### Experimental procedure

Participants did not know about the content of the separate sections of the experiment at the beginning of the experiment but were informed about the subsequent tasks after the completion of each section. However, participants were aware of the content of the individual tasks within each section before submitting their decisions. For example, when reporting their beliefs about the expected behavior in the DG, participants knew that they are also requested to report their beliefs concerning UG behavior.

Half of the participants played the DG, then the UG, followed by the psychometric tests; the remaining participants completed the psychometric tests first and then played the games. We did not find any order effects between the psychometric tests and games. We therefore have pooled the data. Subsequently, participants indicated their beliefs about the likely actions of others in the games. This was followed by measuring participants' risk attitude. Questions on the demographic background of each participant ended the experiment. Participants received their earnings in cash immediately after the completion of the experiment.

When analyzing the data we exclude from the dataset four non-native German speakers, who indicated limited ability to fully respond to the verbal descriptions of emotional states used in one of the psychometric tests. Furthermore, the data reveal that 13 participants reported non-monotonic risk preferences. These individuals are excluded from the analysis when analyzing the impact of risk aversion on participants' decisions and belief formation.

## Results

We find that the mean offer is 25 percent of the endowment in the DG and 40 percent of the endowment in the UG. Modal offers in both games are equally high at 44 percent of the endowment. The mean of the minimum acceptance level in the UG is 26 percent of the endowment and the mode is 33 percent of the endowment. These observations suggests that people in our experiment display a substantial degree of pro-social behavior and reflect the common findings in the literature [9].

Does a greater capacity for empathy result in higher offers (Hypothesis 1)? We find that the two empathy measures are highly convergent (IRI-Empathy and MET-Direct empathy, *r = 0.59*, *p*<.01, for a table showing correlations between all psychometric tests see Table S1) and estimate separate regression models for each empathy measure as depicted in Table 1. The data show no significant relationship between the empathy measures and offers in the DG or UG. Likewise, various alternative behavioral indexes of altruism (e.g., the UG offer minus the UG belief about the minimum acceptance level, the belief about the DG offer by fellow participants minus the DG offer) do not uncover any statistically significant relation.

**Table 1:** Determinants of the Dictator and Ultimatum Game offers – OLS regression.

	DG offers			UG offers		
	Reg. 1 DG	Reg. 2 DG	Reg. 3 DG	Reg. 1 UG	Reg. 2 UG	Reg. 3 UG
IRI - empathy	−0.01			0.14		
	(0.31)			(0.20)		
MET - direct empathy		−1.67			−0.74	
		(1.33)			(0.86)	
MET - indirect empathy			−1.62			−0.61
			(1.35)			(0.87)
IRI - cold ToM	0.50	0.58*	0.60*	0.07	0.17	0.17
	(0.35)	(0.32)	(0.33)	(0.22)	(0.21)	(0.21)
MET - hot ToM	0.33	0.47	0.41	−0.10	0.01	−0.03
	(0.95)	(0.95)	(0.94)	(0.61)	(0.61)	(0.61)
Risk aversion	1.23	1.11	1.08	1.48**	1.45**	1.45**
	(1.14)	(1.14)	(1.14)	(0.73)	(0.73)	(0.74)
Constant	−0.29	4.96	4.49	23.75***	27.10***	26.57***
	(14.41)	(14.74)	(14.70)	(9.24)	(9.52)	(9.50)
N =	103	103	103	103	103	103
F(4, 98) =	1.00	1.41	1.37	1.25	1.32	1.25
Prob>F =	0.41	0.24	0.25	0.30	0.27	0.29
R-squared =	0.04	0.05	0.05	0.05	0.05	0.05

Notes: OLS regression estimates. Standard error in parenthesis.

*** Significant at the 1% level;

** Significant at the 5% level.

We find that individual differences in Cold-ToM as measured by the Interpersonal Reactivity Index do weakly correlate with the DG offers. This finding is in line with previous studies on non-strategic social tasks that have found a positive relationship between pro-social behavior and ToM [37], [38]. A potential explanation suggested by the previous studies is that the origins of human social intelligence lie in the need for cooperation. In this case, people who aim to be fair also need to have a high functioning ToM. At the same time, the lack of monetary incentives in both studies might have not provided an adequate test-bed for assessing the role of ToM among selfish individuals. Notably, we find that risk aversion as measured by the Holt-Laury lottery task significantly and positively influences UG offers, suggesting that strategic considerations in the UG are moderated by individual differences in risk preferences.

An important question from both a behavioral and a methodological perspective is whether greater capacity for ToM elicited using the psychometric scales relates to higher accuracy in the stated beliefs (Hypothesis 2). The results in Table 2 are surprising. In particular, cold ToM as measured through the psychometric scales does not relate to ToM as measured by the accuracy of stated beliefs. At the same time, while the accuracy of beliefs can in principle be influenced by differences in risk attitudes, we find no support for this conjecture. Correlation between the accuracy of beliefs and risk aversion is statistically insignificant for all three measures of accuracy (DG and UG offers, minimum acceptance level in the UG: r<.22, *p*>.12).

**Table 2:** The relationship between ToM and accuracy of beliefs in games.

	IRI - cold ToM	MET - hot ToM
Accuracy of beliefs, DG offer	0.64	−1.50
	(3.23)	(1.07)
Accuracy of beliefs, UG offer	−1.14	−0.31
	(3.18)	(1.06)
Accuracy of beliefs, UG min. acceptance	−1.75	−0.15
	(2.90)	(0.96)
Risk aversion	−0.22	0.14
	(0.38)	(0.12)
Constant	29.55***	13.60***
	(5.22)	(1.73)
N =	103	103
F(4, 98) =	0.29	0.98
Prob>F =	0.88	0.42
R-squared =	0.01	0.04

Notes: OLS regression estimates. Standard errors are presented in parenthesis.

***Significant at 1%.

A possible explanation for the lack of a relationship between the accuracy of beliefs and psychometric ToM measures is that a neutrally framed laboratory experiment is a context in which many of the cues that psychometric tests rely on are stripped away. Even the IRI measure of ToM which is based on abstract description of social interaction likely does not relate well to the simple set-up of games. Yet, the present laboratory setting provides a very specific measure of how good a participant is in judging the action of others which closely relates to ToM: participants stated what they belief others do in the games and were rewarded for their accuracy. The resulting score of accuracy of beliefs then is a laboratory specific measure in how far ToM might inform action.

To test our third hypothesis that fair participants have more accurate beliefs than selfish participants, we use a mean split of the DG offers to distinguish between fair and selfish participants. Alternative definitions of selfishness (e.g., median split or offering nothing) yield qualitatively very similar results to those obtained with the mean split applied here. Note that we do not find evidence to support the conjecture that participants' risk aversion is associated with the dispersion of reported beliefs. This result is robust to different measures of statistical dispersion (variance and kurtosis).

Similarly, behavior in the three belief elicitation tasks is not affected by risk aversion. To address the issue of potential risk hedging between the three belief elicitation tasks we compute various measures for statistical dispersion (variance and kurtosis) and test whether there are systematic differences in reported distributions between the tasks. Our results show that the measures of statistical dispersion remain fairly stable between the tasks. Correlation coefficients between the variances of distributions are as follows: DG and UG-Proposer: 0.52; DG and UG-responder: 0.57; UG-Proposer and UG-responder: 0.60. Correlation coefficients between the kurtoses of distributions are as follows: DG and UG-Proposer: 0.36; DG and UG-responder 0.38; UG-Proposer and UG-responder: 0.45. The used data set excludes four non-native German speakers and 13 individuals who reported non-monotonic rick preferences in our test for risk aversion. In addition, we exclude two individuals who reported uniform belief distributions with a variance of 0 and undefined kurtosis. See Table S2 for the complete table and a comparison between fair and selfish participant (Table S3) where the data reveals no significant difference. This leads us to conclude that the participants do not systematically balance their reported beliefs between these tasks. It should be acknowledged, however, that this does not entirely rule out the possibility that some individuals might have been hedging at the individual level.

The accuracy of beliefs is, as described above in the Behavioral measures section, measured by participants' ability to assess the behavior of fellow participants. The scores for each of the three tasks and test statistics are summarized in Table 3. In support of our third hypothesis, comparing beliefs of fair and selfish participants, we find that fair participants evince a higher accuracy of beliefs about offers made in the DG and UG compared to selfish participants. However, note that there is no difference between fair and selfish participants in the accuracy of beliefs concerning the minimum acceptance level in the UG. Being able to estimate this well would be of particular relevance for selfish participants who seek a high payoff.

**Table 3:** Mean accuracy of beliefs across all three tasks.

	Fair Mean (SD)	Selfish Mean (SD)	MWU	p
DG offer	1.07 (.12)	.95 (.20)	1050.5	<.01
UG offer	1.23 (.11)	1.14 (.21)	1259	<.01
UG min. acceptance	1.05 (.15)	1.05 (.21)	1484.5	0.28
TOTAL	1.12 (.13)	1.04 (.21)	1127.5	<.01

Notes: SD = Standard deviation. MWU = Mann-Whitney U, and two-tailed asymptotic *p* values are shown. Including the total score for selfish (N = 58) and fair (N = 58) participants.

Do individuals capitalize on their accuracy of beliefs in the UG (Hypothesis 4)? To estimate the expected monetary earnings for each participant from the UG, we first calculate how often a participant's offer in the UG is accepted by comparing a participant's UG offer with each of the 115 decisions about the minimum acceptance level made by the responders. We label this value a participant's expected acceptance rate. By multiplying the expected acceptance rate with the UG offer, we compute expected monetary earnings for each participant.

Models 1 and 2 in Table 4 show how selfish and fair participants employ their accuracy of beliefs in the UG. We find that the accuracy of beliefs is strongly associated with larger expected earnings among selfish individuals, whereas there in no such relationship among fair individuals. The result is robust to controlling for risk aversion and demographic variables. The finding suggests that selfish participants effectively utilize ToM in service of their own material goals. In other words, the higher their accuracy of beliefs, the higher their expected earnings.

**Table 4:** Determinants of expected earnings in the UG among selfish and fair individuals - OLS regression.

	Expected earnings UG	
	Selfish	Fair
Accuracy of beliefs, UG min. acceptance	27.15***	−2.55
	(5.51)	(3.45)
IRI - cold ToM	−0.26	0.08
	(0.26)	(0.12)
MET - hot ToM	0.33	−0.43
	(0.73)	(0.32)
Risk aversion	−0.01	0.14
	(0.97)	(0.39)
Constant	17.34	51.57***
	(12.65)	(6.33)
N	51	52
F (4,46/47)	6.39	0.7
Prob>F	<.01	0.6
R^2^	0.36	0.06

Notes: OLS regression estimates. Standard errors are presented in parenthesis.

***Significant at 1%.

## Discussion and Concluding Remarks

Individual differences in empathy and Theory of Mind (ToM) have been hypothesized to underlie differences in social preferences as observed in neutrally framed laboratory games. The Dictator (DG) and Ultimatum Game (UG), feature particularly prominently as measures to elicit such preferences. Indeed, as Kirman and Teschl (2010, p. 311), state “it would, of course, be very convenient if individuals had a certain fixed level of empathy which was independent of the context in which they found themselves in” [15]. Individual differences in empathy as measured by psychometric scales have been found in a number of instances where pro-social behavior is observed, such as volunteering, donating and helping someone in need. These observations lend support to the empathy-altruism hypothesis [19]. Neuroscientific evidence also points in this direction: Empathy-related brain activity is observed in participants when another person who had previously acted in a fair manner receive a painful stimulus [26]. Here we show that contrary to what has been hypothesized in the literature [4], [15], [16], [27], [28] in neutrally framed games individual differences in empathy do not correlate with pro-social behavior. However, we do not claim that these findings necessarily extend to socially framed conditions.

An important element that the above-cited examples have in common highlighting the relationship between empathy and pro-social behaviour is that they resemble situations where another person can be perceived as needy. Indeed, moving from a neutral context to portraying someone in need increases the pro-social behavior of participants [22]. However, this still leaves open why social preferences are observed in neutrally framed laboratory games.

A different route linking social preferences to individual differences has been suggested in the research on ToM. Specifically, cold ToM is defined as the capacity to make inferences regarding the epistemic state of others. Forming beliefs what others are likely to do reflects exactly this capacity. Intriguingly, accuracy of beliefs and the psychometric measures specifically for the capacity for cold ToM are not correlated. A likely reason for this is that the psychometric ToM measures are not able to capture the cues that people respond to in the games. However, ToM as revealed by the accuracy of beliefs strongly relates to the games. The literature suggests two different functions, i) ToM can be applied to pursue one's own material payoff; ii) ToM can facilitate pro-social behavior. We show beliefs in the games have the two functions that the literature ascribes to ToM. Fair participants have more accurate beliefs about the offers made in the DG and the UG compared to selfish participants. However, in the context where it is functional for selfish participants to have accurate beliefs, such as in the role of proposer in the UG, the difference between fair and selfish participants vanishes. In fact, accuracy of beliefs about the responder's minimum acceptance level positively affects earnings for selfish but not fair participants. This suggests individual differences in how accurately people can predict the behavior of others, which conventionally is understood as a feature of the capacity for ToM, play out differently depending on the social preferences that people harbor. Yet, where do these differences originate from that lead people to pursue diverging objectives?

A potential source are personality traits which, akin to individual differences in capacities, are measured using psychometric scales, are fairly stable in an adult, and are considered an important factor in shaping decisions [57]. Using the Big Five [58], the most widely used personality measure, out of the five traits only extraversion was found to have a weak positive relation with offers in the DG [59]. However, using the Myers-Briggs Type Indicator [60] offers in DG and UG did not correlate with extraversion, introversion, or individual differences in perception or judgment [61]. Using the same psychometric scale, a further study finds that extraverts have a somewhat lower minimum acceptance level [62]. Taken together, the research on personality traits suggests that there is some conflicting evidence regarding a connection with social preferences.

Considering the psychometric scales for ToM applied here we find a weak positive correlation between cold ToM, which tests for inferences with regard to the epistemic state of others, and offers in the DG. We find no correlation to hot ToM, which relates to inferences about others' emotions [54]. A possible explanation for this is that social emotions, induced, for instance, by the neediness of someone involved, did not feature in our neutral experiments. The weak positive correlation between DG offers and cold ToM has also been found in other non-strategic settings and has been suggested to be due to the fact that ToM was employed by participants who acted more fairly to match social expectations [37], [38]. Concerning a strategic setting, a positive correlation between ToM and offers has only been found in studies with children [39], [40]. In contrast to our study, these games were not played anonymously but an experimenter was paired with each child and was present at all times. Such a design can invite a demand effect where participants want to appear fair. The importance of social expectations and subtle demand effects on behavior as observed in the DG and UG has been pointed out by a number of studies. For instance, when proposers in the DG are given the option to opt out by accepting a payoff that is lower than what they could gain in the game, about 40 percent of participants opt out. This leaves the receiver without payoff and ensures that she never knows that a DG has been played [47], [63]. Such an effect is present in a number of studies on the DG and UG which find that greater anonymity or the possibilities to obscure the role of the proposer decreases offers [64]–[67]. Future research is needed to specify the exact relationship between demand effect and individual differences in ToM.

Expectations or beliefs also feature in studies that suggest that behavior in neutrally framed DGs and UGs can substantially vary with the environment people live in. When observing the action of others, people form beliefs about what others are likely to do and commonly adopt similar behavior [68]. Comparing different student populations shows that the longer a student has studied economics the lower are offers in the DG whereas those studying social work maintain relatively high offers in the DG throughout their studies [69]. Manipulating the beliefs what others do can cause large shifts in DG and UG offers and the minimum acceptance level in accordance to what the perceived majority choice in the group is [68], [70], [71]. This suggests that beliefs have an important role in games which are commonly used to assess people's social preferences. Similarly, we show that fair and selfish participants differ in their beliefs about what others do and also employ their skills about forecasting what others do differently. Future research would need to address whether a possible important source of observed individual differences in social preferences as observed with a conventional pool of participants can be due to the different environments that people come from when attending laboratory experiments.

## Supporting Information

Instructions S1 Experimental Instructions.
(DOCX)

Table S1 Correlations between all applied psychometric tests.
(DOCX)

Table S2 Correlations between risk aversion and the dispersion of reported belief distributions.
(DOCX)

Table S3 Risk aversion and the dispersion of reported belief distributions in fair and selfish subsamples.
(DOCX)
