# Supplementary material for: In Others' Shoes: Do Individual Differences in Empathy and Theory of Mind Shape Social Preferences?
Source: PLoS One. 2014 Apr 17;9(4):e92844. doi: 10.1371/journal.pone.0092844 (PMC3990498; doi:10.1371/journal.pone.0092844)
Supplement: Instructions S1 — Experimental Instructions. (DOCX) [file pone.0092844.s001.docx]

Experimental Instructions S4

Instructions

(English translation of the original version written in German)

Thank you for coming! You are now about to take part in an experiment on decision making. By reading carefully the following instructions and taking part in the experiment you can earn money depending both on your own decisions and on the decisions of others. During the experiment, you are not allowed to communicate in the laboratory or with someone outside the laboratory. Please switch off your mobile phone. Any violation of these rules will lead to your exclusion from the experiment and no payments. If you have any questions regarding the rules or the course of this experiment, please raise your hand. An experimenter will assist you privately.

This experiment consists of one computerized questionnaire and three separate sections with varying decision tasks. Answering carefully all items in the questionnaire will earn you 4€. In each of the three separate sections, one randomly chosen decision determines your earnings from the section. Your overall income from the experiment will be based on the sum of earnings from the three separate sections and the questionnaire. Neither during nor after the experiment will you or any other participant be informed about the identity of a person with whom you are interacting. Your earnings will be paid privately in cash at the end of the experiment.

During the experiment, all decisions are made in Experimental Currency Units (ECUs). Your total income will be calculated in ECUs and at the end of the experiment converted to Euros at the following rate:

**1 ECU = 0.15€**

The experiment begins with the questionnaire. You have one decision to be taken per computer screen. Please bear in mind that after the introductory stage of two computer screens you have up to 15 seconds to make your decision on each screen. The remaining time is displayed on your screen in the upper right hand corner.

*First section*

The first section consists of two decision tasks in which your earnings depend both on your own decisions and one randomly chosen participant. There are two types of individuals: Type A and type B. You will make a decision in both roles. To calculate your earnings from this section, only one decision will be randomly chosen. The random decision is determined by the computer at the end of the experiment.

First Decision Task

There are two types of individuals: Type A and type B. Person A decides how to divide a pie of 90 ECUs between him/herself and a person B. Person B is passive in this situation. The division is possible only in intervals of 10 currency units. In other words, person A can allocate 0, 10, 20, 30, 40, 50, 60, 70, 80, or 90 ECUs to person B.

**Example:** Person A allocates 30 ECUs to person B, person A earns (90 - 30 = ) 60 ECUs.

Second Decision Task

Person A decides how to divide a pie of 90 ECUs between him/herself and person B. Person B may now either accept or decline the proposed division. Should person B accept the division, both persons earn ECUs in compliance with the proposed division. Should person B decline the offered allocation, both persons earn nothing. To determine the final allocation from the second decision task, person B indicates the minimum amount of ECUs that he/she is willing to accept. Person A's decision to divide the pie and B's minimum that he/she is willing to accept are only possible in intervals of 10 currency units. You are now asked to make your decision in both roles: as person A and B. Your payoff relevant decision will be randomly determined by the computer at the end of the experiment.

**Example:** Person A decides to offer 30 ECUs to person B and thereby keeps 60 ECUs for him/herself. Person B indicates the minimum amount of ECUs he/she is willing to accept. Should the amount be smaller or equal to 30 ECUs, receives person B 30 ECUs and person A 60 ECUs. Should the acceptable amount be greater than 30 ECUs, do both persons receive 0 ECUs from the decision task.

*Second section (This part of the instructions were handed to participants only after finishing the first section).*

The second section consists of three decision tasks that are described below. In this section, your earnings depend both on your own decisions and on the decisions of others. At the end of the experiment, the computer will randomly determine one of the three tasks that will be used to determine your earnings from the second section.

In the first stage there were three situations in which 90 ECUs were at stake:

1. Person A allocates ECUs between him/herself and person B, B is passive;
2. Person A allocates ECUs between him/herself and person B, B is active;
3. Person B indicates the smallest amount that he/she is willing to accept.

All decisions were to be made in intervals of 10 ECUs.

In the following three decision tasks, your earnings will be determined by the accuracy of your probability assessment. Your task is to indicate the likelihood that a randomly determined person has chosen one of the ten possible divisions / minimum acceptance of ECUs. Please note that the sum of your probability assessments needs to equal 100 per cent. Your earnings will be calculated based on the following figure. A more detailed explanation will follow.

| **Stated probability (in percent)** | **Choice of partner correctly predicted (in ECU*)** | **Costs for giving probabilities to not chosen action (in ECU*)** |
| --- | --- | --- |
| 100% | 20.00 ECU | 10.00 ECU |
| 95% | 19.98 ECU | 9.03 ECU |
| 90% | 19.90 ECU | 8.10 ECU |
| 85% | 19.78 ECU | 7.23 ECU |
| 80% | 19.60 ECU | 6.40 ECU |
| 75% | 19.38 ECU | 5.63 ECU |
| 70% | 19.10 ECU | 4.90 ECU |
| 65% | 18.78 ECU | 4.23 ECU |
| 60% | 18.40 ECU | 3.60 ECU |
| 55% | 17.98 ECU | 3.03 ECU |
| 50% | 17.50 ECU | 2.50 ECU |
| 45% | 16.98 ECU | 2.03 ECU |
| 40% | 16.40 ECU | 1.60 ECU |
| 35% | 15.78 ECU | 1.23 ECU |
| 30% | 15.10 ECU | 0.90 ECU |
| 25% | 14.38 ECU | 0.63 ECU |
| 20% | 13.60 ECU | 0.40 ECU |
| 15% | 12.78 ECU | 0.23 ECU |
| 10% | 11.90 ECU | 0.10 ECU |
| 5% | 10.98 ECU | 0.03 ECU |
| 0% | 10.00 ECU | 0.00 ECU |

Note: * ECU stands for Experimental Currency Unit

The payoff consequences of your choices will be explained through the following example: Assume a situation in which person A decides how to allocate a pie of 90 ECUs between him/herself and a person B. Person B is passive. The first column in the table contains the probability that you want to assign for a certain possible division. Should you for instance assess that all 10 possible divisions (from 0 ECUs to 90 ECUs) are equally likely to occur, your decision is to set 10 per cent probability to all possible events.

The second column in the table indicates your earnings from a correct prediction given your probability assessment. In the example, all 10 possible divisions received a probability estimate of 10 per cent. You have inevitably made a correct prediction, which earns you 11.90 ECUs.

You have to bear the costs from incorrect probability assessments (third column). In this example, you have set 10 per cent probability also for all the events that did not occur. These incorrect predictions are all associated with a deduction 0.10 ECUs as can be read from the third column in the table.

That is, your total earnings from the task are 11.90 ECU - 0.10 ECU - 0.10 ECU - 0.10 ECU - 0.10 ECU - 0.10 ECU - 0.10 ECU - 0.10 ECU - 0.10 ECU - 0.10 ECU = 11.90 ECU -- 9 * 0.10 ECU = 11.00 ECU.

Further example: Assume that you have made following probability assessments: 10% for 0 ECUs, 20% for 10 ECUs, 40% for 20 ECUs and 15% each for 30 and 40 ECUs.

The randomly chosen person A decides to allocate 10 ECUs to person B. Your probability assessment for that event was 20%. Your earnings from the decision task will be calculated as following: 13.60 ECUs (20% for a correct prediction) - 0.10 (10% for an incorrect prediction) - 1.60 ECUs (40% for an incorrect prediction) - 2 * 0.23 ECUs (two times 15% for an incorrect prediction) = 11.44 ECUs.

Please pay attention to the fact that under the given payoff scheme the worst possible monetary outcome happens when you set 100 per cent probability for an event that does not occur. Your earnings in such case would be 0 ECUs. On the contrary, should you set 100 per cent probability for an event that occurs, your earnings would be the highest possible with 20 ECUs. Note that you are not bound to make your probability assessments in intervals of 5 percent. This limitation was used only for an illustration. That is, you can for instance set a probability of 97% for a certain event. You will receive a complete payoff table once we begin the experiment.

Training Period (This information and question were originally shown only on the computer screen)

Next we will ask you questions regarding the decision situation. The questions will help you to understand the calculation of your payoff and ensure that you have understood the instructions. Once you have found the correct answer, please insert the value in the given box.

When you have answered both questions correctly, we are ready to begin the experiment. Please answer the following questions with the help of the above given table.

1. You are expecting with a probability of 55% that your partner will give you 30 ECUs. Your partner decides to actually give you 30 ECUs. How many ECUs will you receive for this correct prediction?

2. You are expecting with a probability of 50% that your partner will decide to give you 30 ECUs, with a probability of 30% 20 ECUs, and with a probability of 20% that he will decide to give you 10 ECUs. Your partner decides to actually give you 40 ECUs. How high are your costs (in ECUs) for this incorrect assessment?

3. You are expecting with a probability of 50% that your partner will decide to give you 30 ECUs, with a probability of 30% that he will give you 40 ECU, with a probability of 10% that he will give you 20 ECUs, with a probability of 5% that he will give you 10 ECUs, and with a probability of 5% that he will give you 50 ECUs. Your partner decides to actually give you 30 ECUs. How high is your payoff (in ECUs)?

*Third section [Holt and Laury risk elicitation] (This part of the instructions were handed to participants only after finishing the second section).*

In the following decision situation you can earn money solely depending on your own decisions. Your task is to make a decision in 10 different situations between option A and option B. Each option contains two outcomes (earnings) and probabilities that an outcome will occur.

In order to determine your payoff from this section, the computer will roll two different dices. The first roll with a 10-sided dice determines one of the 10 situations you will get paid for. The second roll with a 20-sided dice will determine which one of outcomes is realized.

Example:

|  | **Option A** | |  | **Option B** | |
| --- | --- | --- | --- | --- | --- |
|  |  | Earnings |  |  | Earnings |
| 1. | If the outcome of the throw is 1 | 20.00 ECUs |  | If the outcome of the throw is 1 | 38.50 ECUs |
|  | If the outcome of the throw is 2-10 | 16.00 ECUs |  | If the outcome of the throw is 2-10 | 1.00 ECUs |

Assume that the first roll of a 10-sided dice results in a 1, hence the first of the ten decisions you made would be selected for payment. Assume that you have chosen the option A. If rolling the 20-sided dice yields a 1, you will earn 20.00 ECUs. If it yields any number between 2 and 10, you will earn 16.00 ECUs. By contrast, if you have chosen option B and rolling the 20-sided dice yields a 1, you will earn 38.50 ECUs; if the dice yields any number between 2 and 10, you will earn 1.00 ECUs.

Your task is now to make a decision between options A and B in all 10 different situations, but only one of them will be used in the end to determine your earnings. Obviously, each situation has an equal chance of being selected in the end. Your earnings are paid accordingly based on your choice in this situation.

The ten situations look as follows:

|  | **Option A** | |  | **Option B** | |
| --- | --- | --- | --- | --- | --- |
|  | Probability  (outcome of the thrown die) | Earnings |  | Probability  (outcome of the thrown die) | Earnings |
| 1. | 1 | 20.00 ECUs |  | 1 | 38.50 ECUs |
|  | 2 - 10 | 16.00 ECUs |  | 2 - 10 | 1.00 ECUs |
|  |  |  |  |  |  |
| 2. | 1 - 2 | 20.00 ECUs |  | 1 - 2 | 38.50 ECUs |
|  | 3 - 10 | 16.00 ECUs |  | 3 - 10 | 1.00 ECUs |
|  |  |  |  |  |  |
| 3. | 1 - 3 | 20.00 ECUs |  | 1 - 3 | 38.50 ECUs |
|  | 4 - 10 | 16.00 ECUs |  | 4 - 10 | 1.00 ECUs |
|  |  |  |  |  |  |
| 4. | 1 - 4 | 20.00 ECUs |  | 1 - 4 | 38.50 ECUs |
|  | 5 - 10 | 16.00 ECUs |  | 5 - 10 | 1.00 ECUs |
|  |  |  |  |  |  |
| 5. | 1 - 5 | 20.00 ECUs |  | 1 - 5 | 38.50 ECUs |
|  | 6 - 10 | 16.00 ECUs |  | 6 - 10 | 1.00 ECUs |
|  |  |  |  |  |  |
| 6. | 1 - 6 | 20.00 ECUs |  | 1 - 6 | 38.50 ECUs |
|  | 7 - 10 | 16.00 ECUs |  | 7 - 10 | 1.00 ECUs |
|  |  |  |  |  |  |
| 7. | 1 - 7 | 20.00 ECUs |  | 1 - 7 | 38.50 ECUs |
|  | 8 - 10 | 16.00 ECUs |  | 8 - 10 | 1.00 ECUs |
|  |  |  |  |  |  |
| 8. | 1 - 8 | 20.00 ECUs |  | 1 - 8 | 38.50 ECUs |
|  | 9 - 10 | 16.00 ECUs |  | 9 - 10 | 1.00 ECUs |
|  |  |  |  |  |  |
| 9. | 1 - 9 | 20.00 ECUs |  | 1 - 9 | 38.50 ECUs |
|  | 10 | 16.00 ECUs |  | 10 | 1.00 ECUs |
|  |  |  |  |  |  |
| 10. | 1 - 10 | 20.00 ECUs |  | 1 - 10 | 38.50 ECUs |
