# Supplementary material for: In Others' Shoes: Do Individual Differences in Empathy and Theory of Mind Shape Social Preferences?
Source: PLoS One. 2014 Apr 17;9(4):e92844. doi: 10.1371/journal.pone.0092844 (PMC3990498; doi:10.1371/journal.pone.0092844)
Supplement: Table S1 — Correlations between all applied psychometric tests. (DOCX) [file pone.0092844.s002.docx]

Table S1: Correlations between all applied psychometric tests.

|  | (1) | (2) | (3) | (4) | (5) |
| --- | --- | --- | --- | --- | --- |
| Variables |  |  |  |  |  |
| IRI – Empathy | 1 |  |  |  |  |
| IRI – ToM | .675*** | 1 |  |  |  |
| MET – Direct Empathy | .552*** | .257*** | 1 |  |  |
| MET – Indirect Empathy | .544*** | .297*** | .923*** | 1 |  |
| MET - ToM | .290*** | .226*** | 0.177 | 0.141 | 1 |

Notes: IRI = Interpersonal Reactivity Index, MET = Multifaceted Empathy Test, ToM = Theory of Mind. N=116. ***Significant at 1% level (two-tailed).
