# Supplementary material for: In Others' Shoes: Do Individual Differences in Empathy and Theory of Mind Shape Social Preferences?
Source: PLoS One. 2014 Apr 17;9(4):e92844. doi: 10.1371/journal.pone.0092844 (PMC3990498; doi:10.1371/journal.pone.0092844)
Supplement: Table S2 — Correlations between risk aversion and the dispersion of reported belief distributions. (DOCX) [file pone.0092844.s003.docx]

Table S2: Correlations between risk aversion and the dispersion of reported belief distributions

|  | (1) | (2) | (3) | (4) | (5) | (6) | (7) |
| --- | --- | --- | --- | --- | --- | --- | --- |
| Variables |  |  |  |  |  |  |  |
| Risk aversion (Holt and Laury) | 1 |  |  |  |  |  |  |
| Kurtosis DG-Belief | -.03 | 1 |  |  |  |  |  |
| Kurtosis UG-Proposer-Belief | -.12 | .36*** | 1 |  |  |  |  |
| Kurtosis UG-Responder-Belief | .01 | .38*** | .45*** | 1 |  |  |  |
| Variance DG-Belief | -.08 |  |  |  | 1 |  |  |
| Variance UG-Proposer-Belief | -.16 |  |  |  | .52*** | 1 |  |
| Variance UG-Responder-Belief | -.13 |  |  |  | .57*** | .60*** | 1 |

Notes: Pearson's correlation coefficients, N=101. The applied data set excludes four non-native German speakers and 13 individuals who reported non-monotonic rick preferences in our test for risk aversion. In addition, we exclude two individuals who reported uniform belief distributions with a variance of 0 and undefined kurtosis. ***Significant at 1% level (two-tailed). DG = Dictator Game, UG=Ultimatum Game
