# Supplementary material for: In Others' Shoes: Do Individual Differences in Empathy and Theory of Mind Shape Social Preferences?
Source: PLoS One. 2014 Apr 17;9(4):e92844. doi: 10.1371/journal.pone.0092844 (PMC3990498; doi:10.1371/journal.pone.0092844)
Supplement: Table S3 — Risk aversion and the dispersion of reported belief distributions in fair and selfish subsamples. (DOCX) [file pone.0092844.s004.docx]

Table S3: Risk aversion and the dispersion of reported belief distributions in fair and selfish subsamples.

|  | Fair | | | Selfish | | | P-value |
| --- | --- | --- | --- | --- | --- | --- | --- |
| Variables | N | Mean | Std. | N | Mean | Std. |  |
| Risk aversion (Holt and Laury) | 50 | 6.56 | 1.51 | 51 | 6.22 | 1.32 | .23 |
| Kurtosis DG-Belief | 50 | 0.90 | 2.79 | 51 | 1.24 | 3.14 | .57 |
| Kurtosis UG-Proposer-Belief | 50 | 1.70 | 3.02 | 51 | 1.47 | 3.06 | .71 |
| Kurtosis UG-Responder-Belief | 50 | 1.16 | 2.79 | 51 | 2.34 | 3.89 | .09 |
| Variance DG-Belief | 50 | 227.72 | 156.19 | 51 | 229.99 | 136.61 | .94 |
| Variance UG-Proposer-Belief | 50 | 274.04 | 170.83 | 51 | 263.16 | 187.44 | .76 |
| Variance UG-Responder-Belief | 50 | 252.08 | 160.44 | 51 | 306.48 | 217.37 | .16 |

Notes: P-values refer to the significance of (2-tailed) t-tests for differences between fair and selfish participants. DG = Dictator Game, UG=Ultimatum Game. The applied data set excludes four non-native German speakers and 13 individuals who reported non-monotonic rick preferences in our test for risk aversion. In addition, we exclude two individuals who reported uniform belief distributions with a variance of 0 and undefined kurtosis
